# Supplementary material for: An interstellar synthesis of phosphorus oxoacids
Source: Nat Commun. 2018 Sep 21;9:3851. doi: 10.1038/s41467-018-06415-7 (PMC6155066; doi:10.1038/s41467-018-06415-7)
Supplement: Supplementary file 1 — Supplementary Information [file 41467_2018_6415_MOESM1_ESM.pdf]

Supplementary Information

An Interstellar Synthesis of Phosphorus Oxoacids

Turner et al.

Supplementary Table 1 Infrared assignments for phosphine (PH_3) –carbon dioxide (CO_2) ices at 5 K prior to the irradiation, after the irradiation, and of the residues at 300 K.

Assignment	Position with ^{16}O (cm^{-1})	Position with ^{18}O (cm^{-1})	Reference
Pristine ice, before irradiation (5 K)			
CO_2 (ν_2)	651	638	1
PH_3 (ν_2)	985	985	2
PH_3 (ν_4)	1107	1107	2
CO_2 (ν_1)/CO_2 ($2\nu_2$)*	1226, 1336	1276, 1383	1
PH_3 ($2\nu_4$)	---	2207	2
$^{13}CO_2$ (ν_3)	2278	2245	1
PH_3 (ν_1/ ν_3)	2317	2314	2
CO_2 (ν_3)	2325	2292	1
CO_2 ($\nu_3 + \nu_L$)	2398-2440	2360-2390	1
PH_3 ($\nu_1 + \nu_4$)	3428	3428	2
CO_2 ($2\nu_2 + \nu_3$)	3596	3511	1
CO_2 ($\nu_1 + \nu_3$)	3702	3622	1
New peaks after irradiation (5 K)			
Assignment	Position with ^{16}O (cm^{-1})	Position with ^{18}O (cm^{-1})	Reference
ν(P-O)	880-1010	850-950	3
O_3 (ν_3)	1040	975	4
δ(PH_2)	1030	1025	3
ν(P=O)	1180-1370	1150-1340	3
H_2O (ν_2)	1650	1630	5
δ(O=P-OH)	1730	1700	3
ν(C=O)	1780	1750	3
^{13}CO (ν_1)	2090	2042	1
CO (ν_1)	2140	2090	1
P-OH *ν*(OH)	---	2150	3
ν(PH)	2315	2230	3
P_2H_4 (ν_1)	2280	2265	6
P-OH *ν*(OH)	2875	2650-2800	3
H_2O (ν_1(in phase))	2970	2960	5
H_2O (ν_3(transversal))	3230	3200	5
H_2O (ν_3(longitudinal))	3410	3360	5
H_2O (ν_1(out of phase))	3510	3460	5
Residue (300 K)			
Assignment	Position with ^{16}O (cm^{-1})	Position with ^{18}O (cm^{-1})	Reference
ν(P-O)	930-1040	910-1030	3
δ(PH_2)	1080-1115	1050-1115	3
ν(P=O)	1150-1330	1140-1300	3
δ(O=P-OH)	1530-1700	1530-1730	3
P-OH *ν*(OH)	2170	2160	3
ν(PH)	2270	2270	3
P-OH *ν*(OH)	2450-2720	2450-2680	3
ν(OH)	2780-3500	2780-3300	3

Note. ν_L defines the lattice mode.
*Fermi resonance

Supplementary Table 2 Infrared assignments for phosphine (PH_3) – water (H_2O) ices at 5 K prior to the irradiation, after the irradiation, and of the residues at 300 K.

Pristine ice, before irradiation (5 K)			
Assignment	Position with ^{16}O (cm^{-1})	Position with ^{18}O (cm^{-1})	Reference
H_2O (ν_L)	770	740	[5]
PH_3 (ν_2)	983	983	[2]
PH_3 (ν_4)	1101	1103	[2]
H_2O (ν_2)	1650	1640	[5]
PH_3 (ν_1/ ν_3)	2319	2314	[2]
H_2O (ν_1(in phase))	3040	3090	[5]
H_2O (ν_3(transversal))	3220	3250	[5]
H_2O (ν_3(longitudinal))	3380	3375	[5]
H_2O (ν_1(out of phase))	3550	3440	[5]
**New peaks after irradiation (5 K)**			
Assignment	Position with ^{16}O (cm^{-1})	Position with ^{18}O (cm^{-1})	Reference
ν(P-O)	950	970	[3]
δ(PH_2)	1045	1090	[3]
ν(P=O)	1140-1300	1140	[3]
δ(O=P-OH)	1710	1670	[3]
P-OH ν(OH)	2170	2160	[3]
P_2H_4 (ν_1)	2280	2275	[6]
ν(OH)	2900	2910	[3]
**Residue (300 K)**			
Assignment	Position with ^{16}O (cm^{-1})	Position with ^{18}O (cm^{-1})	Reference
ν(P-O)	790-950	780-930	[3]
δ(PH_2)	1040	1050	[3]
ν(P=O)	1120-1320	1100-1300	[3]
δ(O=P-OH)	1550-1670	1530-1640	[3]
ν(PH)	2230	2240	[3]
P-OH *ν*(OH)	2700	2460-2660	[3]
ν(OH)	2950-3000	2750-3300	[3]

Note. ν_L defines the lattice mode.

Supplementary Table 3 SIMS calibration data of pure pyrophosphoric acid, phosphoric acid, and phosphorous acid scaled to the intensity of the most intense signal.

Positive Ions		Negative Ions	
	Pyrophosphoric Acid, $H_4P_2O_7$		
$H_5P_2O_7^+$	17.1	$H_3P_2O_7^-$	45.2
$H_4PO_4^+$	100	$HP_2O_6^-$	93.0
$H_2PO_3^+$	18.9	$H_2PO_4^-$	8.2
$H_4PO_2^+$	5.2	PO_3^-	100
$H_2PO_2^+$	9.8	PO_2^-	16.9
PO_2^+	13.3		
PO^+	10.2		
	Phosphoric Acid, H_3PO_4		
$H_4PO_4^+$	100	$H_2PO_4^-$	23.8
$H_2PO_3^+$	28.1	PO_3^-	100
$H_4PO_2^+$	1.1	PO_2^-	2.0
$H_2PO_2^+$	9.3		
PO_2^+	4.3		
PO^+	20.7		
	Phosphorous Acid, H_3PO_3		
$H_4PO_3^+$	100	$H_2PO_3^-$	17.7
$H_2PO_3^+$	17.5	PO_3^-	100
$H_4PO_2^+$	7.4	PO_2^-	37.8
$H_2PO_2^+$	72.6		
PO_2^+	1.4		
PO^+	72.3		

Supplementary Table 4 SIMS analysis of the residues formed at 5000 nA irradiation showing the raw ion counts and accounting for overlapping ion counts from various oxoacids (corrected ion counts) exploiting the calibration experiments.

	PH_3/CO_2		PH_3/H_2O		$PH_3/C^{18}O_2$		$PH_3/H_2{}^{18}O$	
Positive Ions	Ion Counts ($\times 10^5$)	Corrected Ion Counts ($\times 10^5$)	Ion Counts ($\times 10^5$)	Corrected Ion Counts ($\times 10^5$)	Ion Counts ($\times 10^5$)	Corrected Ion Counts ($\times 10^5$)	Ion Counts ($\times 10^5$)	Corrected Ion Counts ($\times 10^5$)
$H_5P_2O_7^+$	1.8	0	1.4	0	0.22	0	0.07	0
$H_4PO_4^+$	11.5	0.9	9.3	1.0	3.3	21	5.9	5.5
$H_4PO_3^+$	3.3	0	0.9	0	0.4	0	0.3	0
$H_2PO_3^+$	5.7	2.9	6.0	4.0	1.0	0.2	2.6	0.9
$H_2PO_2^+$	6.1	2.6	3.6	2.1	4.7	4.1	10.4	9.6
Negative Ions	Ion Counts ($\times 10^5$)	Corrected Ion Counts ($\times 10^5$)	Ion Counts ($\times 10^5$)	Corrected Ion Counts ($\times 10^5$)	Ion Counts ($\times 10^5$)	Corrected Ion Counts ($\times 10^5$)	Ion Counts ($\times 10^5$)	Corrected Ion Counts ($\times 10^5$)
$H_3P_2O_7^-$	4.0	0	6.1	0	0.3	0	0.3	0
$HP_2O_6^-$	52.8	44.5	29.9	17.4	0.8	0.3	0.1	0
$H_2PO_4^-$	3.0	2.2	5.1	4.0	4.1	4.0	1.6	1.5
$H_2PO_3^-$	1.1	0	0.8	0	0.8	0	0.5	0
PO_3^-	37.9	13.2	35.0	0.5	17.8	0	7.4	0
PO_2^-	9.9	5.8	10.5	6.3	3.8	1.6	1.9	0.5

Supplementary Table 5 Identified phosphorus oxoacids as trimethylsilyl (TMS) derivatives by GC×GC-TOFMS.

**Compound**	R_{t1} [a] [min]	R_{t2} [b] [sec]	MS-fragmentation / ^{18}O sample		MS-fragmentation / ^{16}O standard	
			[$M^{+\bullet}$]	Other important ions, *m/z*	[$M^{+\bullet}$]	Other important ions, *m/z*
Hypophosphorous acid (+I)	12.40	2.7	214	199, 73	210	195, 73
Phosphonic acid (+III)	13.24	1.6	304	289, 207, 147, 133, 75	298	283, 207, 147, 133, 75
Phosphoric acid (+V)[c]	18.59	1.6	322	307, 291, 147, 133, 73, 45	314	299, 283, 211, 147, 133, 73, 45
Phosphoric acid (+V)[d]			320	305, 289, 147, 133, 73, 45		

[a] GC×GC retention time 1st dimension. [b] GC×GC retention time 2nd dimension. [c] [$M^{+\bullet}$] = 322 corresponds to $H_3P^{18}O_4$ and [d] [$M^{+\bullet}$] = 320 to $H_3P^{18}O_3{}^{16}O$.

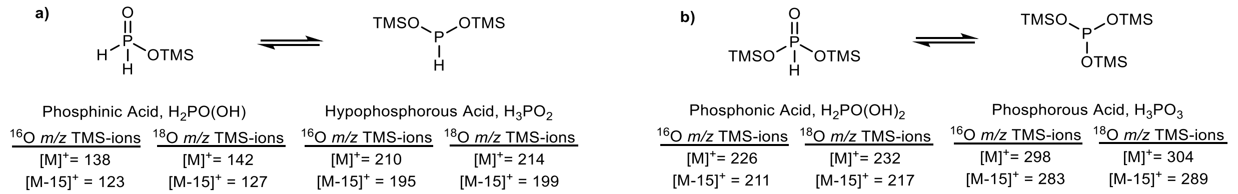

Supplementary Figure 1 Tautomeric forms of trimethylsilyl (TMS) derivatives of H_3PO_2 (a) and H_3PO_3 (b). The characteristic molecular ion peak $[M]^+$ as well as $[M\text{-}15]^+$ of the ^{18}O-TMS derivatives of hypophosphorous acid and phosphorous acid, respectively, revealed the formation of both phosphorus oxoacids in the residues of the irradiated phosphine-doped interstellar ice analogues.

Supplementary Table 6 Calculated enthalpies of formation at 0 K for distinct isomers of phosphorus oxoacids.

Structure	Formula	Name	$\Delta_f H$ (eV)[a]
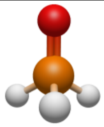	H_3PO	Phosphine oxide	-3.63
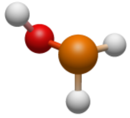	H_2POH	Hydroxyphosphine	-3.60
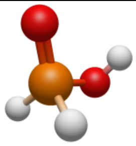	$H_2P(O)OH$	Phosphinic acid	-6.76
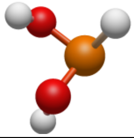	$HP(OH)_2$	Hypophosphorous acid	-6.39
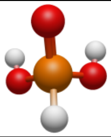	$HPO(OH)_2$	Phosphonic acid	-10.04
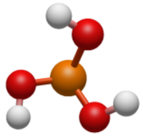	$P(OH)_3$	Phosphorous acid	-9.52
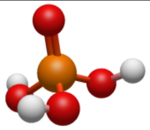	$PO(OH)_3$	Phosphoric acid	-13.13

[a]CCSD(T)/CBS

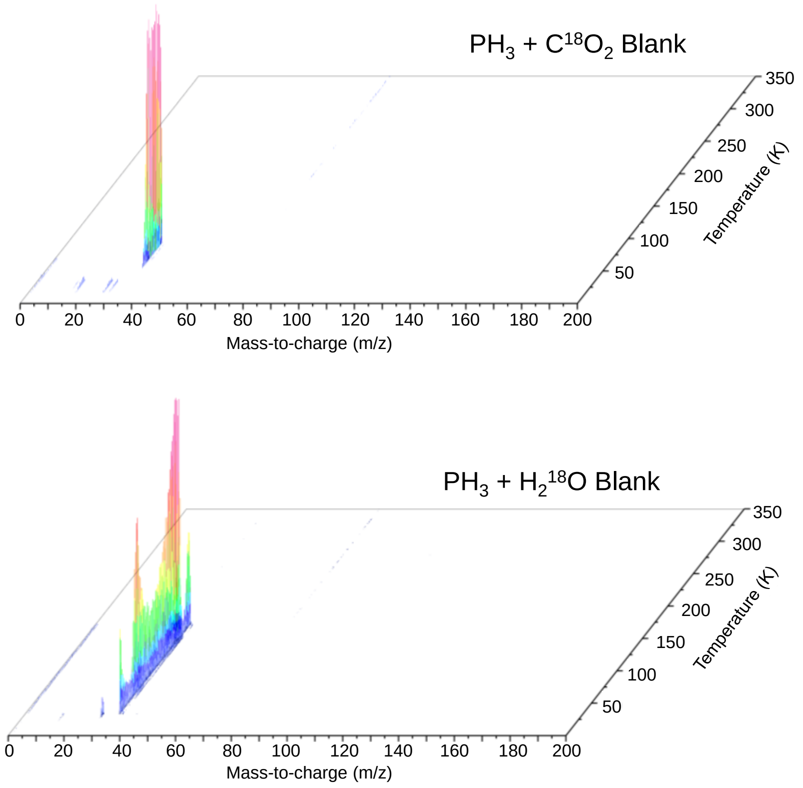

Supplementary Figure 2 PI-ReTOF-MS data as a function of temperature during TPD for the unirradiated ice mixtures of $PH_3/C^{18}O_2$ (top) and $PH_3/H_2{}^{18}O$ (bottom) at 10.86 eV photoionization energy. The strong signal at m/z = 34 results from PH_3, while H_2O and CO_2 have higher ionization energies than 10.86 eV and thus are not observed. It shall be highlighted that no oxoacids are observed in these blank experiments.

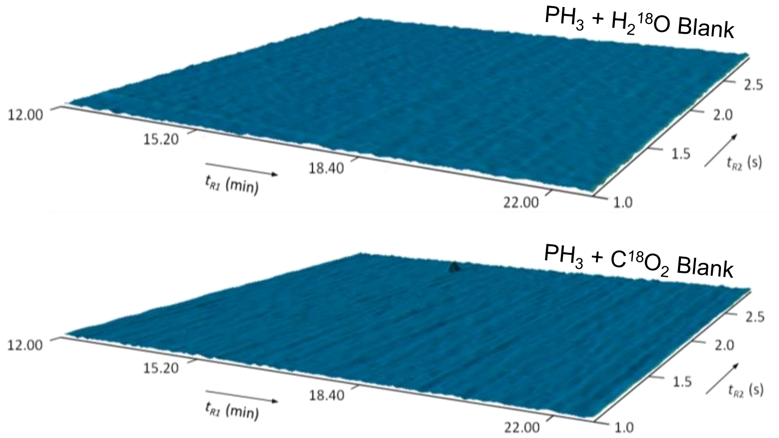

Supplementary Figure 3 GC×GC-TOFMS chromatogram of the blank experiments from the mixtures of $PH_3/C^{18}O_2$ (top) and $PH_3/H_2{}^{18}O$ (bottom). Mass-to-charges (m/z) of 214 (×100) and 304 (×150) were selected for the above representation (*z*-axis = 600,000). It shall be highlighted that no oxoacids are observed in these blank experiments.

Supplementary References

1. Bennett, C. J., Jamieson, C., Mebel, A. M. & Kaiser, R. I. Untangling the formation of the cyclic carbon trioxide isomer in low temperature carbon dioxide ices. *Phys Chem Chem Phys* **6**, 735-746 (2004).
2. Turner, A. M., Abplanalp, M. J. & Kaiser, R. I. Probing the carbon–phosphorus bond coupling in low-temperature phosphine (PH_3)–methane (CH_4) interstellar ice analogues. *Astrophys J* **819**, 97 (2016).
3. Socrates, G. *Infrared and Raman Characteristic Group Frequencies, Third Edition*. (John Wiley & Sons, Ltd., 2004).
4. Ennis, C. P., Bennett, C. J. & Kaiser, R. I. On the formation of ozone in oxygen-rich solar system ices via ionizing radiation. *Phys Chem Chem Phys* **13**, 9469-9482 (2011).
5. Hagen, W., Tielens, A. & Greenberg, J. The infrared spectra of amorphous solid water and ice Ic between 10 and 140 K. *Chem Phys* **56**, 367-379 (1981).
6. Durig, J., Shen, Z. & Zhao, W. Conformational stability, structural parameters, and vibrational frequencies from ab initio calculations for biphosphine. J *Mol Struct: THEOCHEM* **375**, 95-104 (1996).
